# Prevalence and risk factors of *Campylobacter jejuni* and *Campylobacter coli* in fresh chicken carcasses from retail sites in Bogotá, Colombia

**DOI:** 10.1016/j.heliyon.2024.e26356

**Published:** 2024-02-17

**Authors:** Brigithe Tatiana Ortiz, Deyci Rodríguez, Silvia Restrepo

**Affiliations:** a Laboratory of Mycology and Phytopathology (LAMFU), Department of Chemical and Food Engineering, Universidad de los Andes, Bogotá D.C., Colombia; b Laboratory of Food Microbiology, Faculty of Sciences, Pontificia Universidad Javeriana, Bogotá D.C., Colombia

**Keywords:** *Campylobacter jejuni*, *Campylobacter coli*, Colombia, Risk factors, Chicken carcass

## Abstract

*Campylobacter* is one of the most common causes of foodborne gastroenteritis. The objective of this study was to estimate the prevalence and risk factors associated with *Campylobacter jejuni* and *Campylobacter coli* species in fresh chicken carcasses for human consumption from farmers' markets and small food stores in seven localities of Bogotá, Colombia. Ninety-one samples of fresh chicken carcasses were collected from farmers’ markets and small food stores at seven localities in Bogotá. Samples were tested for *Campylobacter* using the real-time polymerase chain reaction (real time PCR) and isolation by plating. To analyze possible risk factors associated with *Campylobacter* spp. contamination in retail chicken carcasses, information was collected using a structured questionnaire and a univariate logistic regression analysis (α = 0.05) was used. Forty-two positive samples were obtained for *Campylobacter* spp., given a prevalence of 46.2%, of which 54.8% were to *C. jejuni*, 9.52% to *C. coli* and 35.7% to joint contaminations. *C. jejuni* was the most prevalent species. Risk factors found included poor cleanliness, in frequency of disinfection, type of establishment, and direct contact of chickens with other food. This study is the first report in the country on the prevalence and risk factors of *Campylobacter* in retail chicken.

## Highlights

• Prevalence of *Campylobacter* in retail chicken carcasses Bogotá (Colombia) was 46.2%.
• Higher *Campylobacter* recovery rate was obtained samples from small food stores.
• *C. jejuni* was more predominant than *C. coli* among isolates from retail chicken.
• Higher proportion of contamination at the simultaneous by both species was evidenced.
• Risk factors were poor hygiene practices, type of establishment and cross-contact.

## 1 Introduction

Foodborne diseases are a significant public health problem worldwide. One of the most important foodborne diseases is *Campylobacter* spp. a bacterial genus considered by the World Health Organization to be on the top four leading global causes of acute diarrheal disease (ADE) and the most frequent gastroenteritis-causing bacterium in the world [1]. *Campylobacter* causes campylobacteriosis in humans and animals and is recognized as a zoonosis of worldwide distribution [2]. *Campylobacter jejuni* and *Campylobacter coli* are the species commonly implicated in human cases, and most infections present as mild or asymptomatic gastroenteritis [1]. However, the infection may lead to severe complications, such as reactive arthritis, Miller-Fisher syndrome (MFS) and Guillain Barré syndrome (GBS) [3]. *Campylobacter* is a Gram-negative bacterium that does not form spores, has a curved or spiral morphology, usually has a slow growth (up to 96 h), and presents a rapid and characteristic dart-like or corkscrew-like movement, as its members possess a polar flagellum at one or both ends of the cell. This microorganism is sensitive to oxygen and free radicals, so they are considered microaerophiles [4,5]. Most *Campylobacter* species are thermotolerant, growing between 37 and 42 °C, with an optimum temperature of 41.5 °C– 42 °C. However, They have shown that they can resist low temperatures <4 °C [6]. In addition, under environmental stress, *Campylobacter* can enter a viable but non-culturable cell state (VBNC), a state in which the bacterium stop growing on commonly used culture media but remains viable with minimal metabolic activity [7,8].

The prevalence of *Campylobacter* worldwide is variable but is usually higher in products whose sources are broiler chicken [9,10]. Poultry is the main reservoir of *Campylobacter* spp. since their intestines provide a favorable environment as a supply of nutrients that support their growth and colonization [11,12]. Once a bird is infected, *Campylobacter* can be transmitted horizontally to most of the other birds in the flock in just a few days, reaching between 10 and 10^8^ CFU/g in the intestinal tract, and remaining colonizing the animal until slaughter [13]. Based on the above, it is considered that one of the main routes of transmission of *Campylobacter* spp. to humans is the consumption of contaminated poultry products, especially by the *C. jejuni* species, which is usually the predominant colonizer, followed by *C. coli* [13,14]. However, these species can also be transmitted through other contaminated foods, such as unpasteurized milk and untreated water [14,15].

Contamination of chicken carcasses can lead to the survival of *Campylobacter* in raw poultry products during their shelf life and generate a risk of possible infection in humans who consume these contaminated products [15]. Likewise, other risk factors such as cross-contamination phenomena through contact with contaminated handlers, surfaces, utensils and food, infected pets, and farm animals, especially in developing countries, are a high risk of transmission to humans [16].

Studies on the percentage prevalence of this pathogen in chicken have been conducted in different parts of the world and variability has been found. For example, in China, 29 chicken carcass samples were analyzed, and a prevalence of 79.5% was detected [17]. In Croatia, 241 fresh chicken samples were analyzed, and 73.9% prevalence for *Campylobacter* spp. was found [18]. However, in countries in the Americas such as Mexico, Argentina and Colombia, there are not many studies and prevalence information available. In a study conducted in Mexico, a prevalence of 89% was found in the analysis of 76 fresh chicken samples [19] and another study in Peru showed a prevalence of 97.5% in the analysis of 120 fresh chicken samples [20].

There exist a European Union Regulation 2017/1495 published in 2017, which incorporates methods for testing of poultry carcasses for *Campylobacter* and establishes a process hygiene criterion for *Campylobacter* in broiler carcasses [21]. Likewise, regulations on campylobacteriosis place it under surveillance in several countries, including in North America, Europe and Asia. For example, human campylobacteriosis is a notifiable disease in Europe [22]. It has been nationally notifiable in Canada since 1986 and in the United States since 2015, it became notifiable at the national level [22,23]. However, in some developing countries, such as Brazil, Mexico, Thailand and the Central African Republic, the lack of routine regulation and diagnosis, there is a problem in analyzing and understanding the importance of campylobacteriosis [24,25]. Some reviews on the global epidemiology of *Campylobacter* spp. show that usually in developing countries, there is no routine diagnosis of this pathogen and no adequate epidemiological surveillance [26].

The poultry industry in Colombia is one of the most dynamic fields in the agricultural sector, especially in broiler production. Colombia ranks 11th in the world in chicken production and 5th in the Americas after the United States, Brazil, Mexico, and Argentina [27]. In terms of per capita consumption, Colombia ranks 14th in the world, with a significant increase over the last 22 years from 14 to 36.3 kg/inhabitant [28]. Among the few studies conducted in Colombia, one developed by the National Institute of Health, known in Colombia as Instituto Nacional de Salud (INS) in 2012, found a prevalence of 10.9% for *Campylobacter* in 19 chicken carcass processing plants [29]. Another study conducted in 2016 found a prevalence of 10% [30]. In addition, the Colombian Integrated Surveillance Program for Antimicrobial Resistance (COIPARS) has identified *Salmonella* spp. and *Campylobacter* spp. as two of the most important foodborne pathogens for public health in the country [[31], [32], [33]]. Despite this, above, there are no studies on *Campylobacter* spp. in chicken carcasses in retail markets and possible risk factors associated with contamination by this pathogen. This may be due to difficulties in its detection in the absence of epidemiological surveillance and mandatory reporting of *Campylobacter* spp. in commercial establishments [29,31,34]. Therefore, the objective of this study was to estimate the prevalence and risk factors associated with *Campylobacter jejuni* and *Campylobacter coli* species in fresh chicken carcasses for human consumption from farmers’ markets and small food stores in seven localities of Bogotá, Colombia.

## 2 Methods

### 2.1 Sample collection

**Fig. 1:**
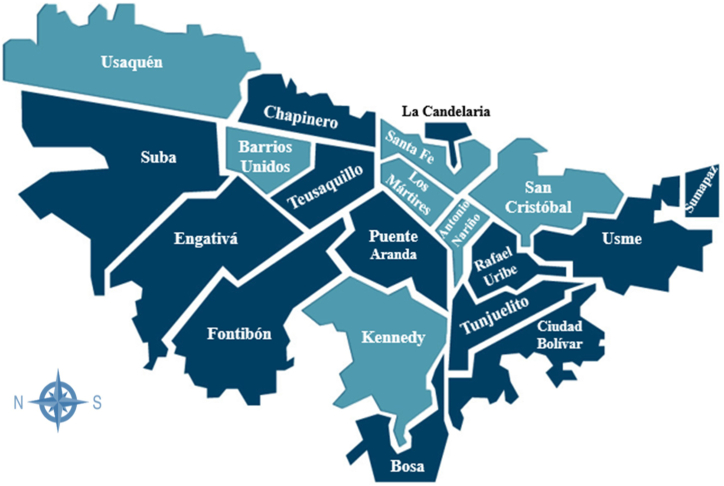
Geographical distribution of the sampled localities of Bogotá: Antonio Nariño, Barrios Unidos, Kennedy, Los Mártires, San Cristóbal, Santa Fe and Usaquén.

Ninety-one samples of fresh chicken carcasses were collected from farmers' markets and retail small food stores located in seven localities of the city of Bogotá through convenience sampling (Fig. 1). The following localities were selected: Antonio Nariño (South), Barrios Unidos (North), Kennedy (Southwest), Los Mártires (East), San Cristóbal (Southeast), Santa Fe (Downtown) and Usaquén (North). For each location, 13 chicken carcass samples (approximately eight from farmers' markets and five from small food stores) were collected between June and November 2021. The chicken carcasses were purchased, therefore, no approval from the Committee on Investigation and Use of Laboratory Animals (CICUAL) was required. At each point of purchase, the internal temperature of the samples was measured with a digital punch thermometer to determine the storage conditions for sale. Each sample was kept in plastic bags, labeled and transported under refrigerated conditions to the Food Microbiology laboratory of the Pontificia Universidad Javeriana where it's were processed on same day.

### 2.2 Questionnaire

At each sampling site, a structured questionnaire approved by the Ethics Committee of the School of Engineering of the Universidad de los Andes was administered to vendors to obtain information on handling, storage, and hygiene conditions to evaluate possible risk factors associated with *Campylobacter* contamination of chicken. Surveyed persons were informed about the procedure on the basis of an informed consent form. The questionnaire included closed-ended questions to collecting data on the following elements: type and location of the site, product storage conditions, cleaning practices and frequency, personal protection elements, and worktop material. Some questions were verified by direct observation by the interviewer (Table S1, Supplementary Material).

### 2.3 Statistical analysis

To analyze risk factors, a descriptive univariate logistic regression analysis was performed using the RStudio program version February 1, 5033 (R Core Team, 2020). However, four variables: i) use of work clothing, ii) type of countertop, iii) use of sanitizers, and iv) how the food was displayed did not elicit different responses among respondents, so they were omitted from the analysis. A conditional logistic model was used for univariate analysis, and exposures with p < 0.05 were considered significant. Variables were constructed by combining multiple items from the questionnaire. Additionally, the Chi-square test using RStudio software was used to test associations with significance between the prevalence of *Campylobacter* spp. in fresh chicken carcasses and sample origin: place/stores and localities. The results were considered statistically significant for p-values < 0.05. In addition, a generalized linear model (GLM) with binomial distribution was performed to show the differences between localities given the prevalence of *Campylobacter* in each one. The GLM used, the bias-reduced logistic regression from the brglm2 package of the RStudio program was used; the results were statistically significant with p-values < 0.1.

### 2.4 Sample processing

Sample processing was performed according to the MLG 41.04 USDA-FSIS protocol "Isolation and identification of *Campylobacter jejuni*/*lari*/*coli* from poultry, sponge and raw product rinse samples" [35]. Each sample was aseptically removed and placed in a pre-labeled WhirlPak® NASCO plastic bag (37.5 × 50 cm) and rinsed with 400 ml of buffered peptonized water (BPW) (Britania SA, CABA, Argentina), manually massaging for 3–5 min to ensure complete contact with the BPW. Then, 30 ml of the rinse was removed, and a selective enrichment was performed in 30 ml of Bolton broth supplemented with antibiotics and 5% horse blood (2X BF-BEB) (Oxoid cat no. SR0117, England) in 100 ml WhirlPak® bags. Enrichments were incubated at 42 °C for 24 h under microaerobiosis conditions using a CampyGen Oxoid™ envelope (CN0025A, Oxoid Ltd, Wade Road, Basingstok, UK) inside a 2.5 L OXOID® vessel.

### 2.5 Detection and identification

Detection and identification *Campylobacter* species was performed according to the recommendations of the MLG 41.04 USDA-FSIS protocol, and samples were analyzed by the real-time polymerase chain reaction assay (real time PCR) using the BAX® System (Hygiena, BAXQ7) kit for *Campylobacter* spp. Described in MLG 41A.00 [35]. Lysis was performed using a ratio of 150 μl of protease per 12,000 μl of lysis buffer and five μl of the previously incubated enrichment. Lysis was performed in a heating block with the following conditions: preheating at 37 °C for 20 min, 95 °C for 10 min. At the end of lysis, the tubes were cooled to 8 °C. Afterwards subsequently 30 μl was taken to hydrate, the PCR tablets in the kit and the real time PCR process was performed in the BAX® equipment for 70 min. *C. jejuni* ATCC® 33560 and *C. coli* ATCC®43478 strains were used for quality control.

### 2.6 Isolation

Isolations were performed in two ways and in parallel to achieve better isolate colonies that met the expected morphology of *C. jejuni* and *C. coli*. For both species, translucent or mucoid colonies were expected, shiny and generally with a faint pink color in the medium, flat, or slightly elevated, which can vary significantly in size [35]. The two plating procedures were performed by filtration and depletion seeding. Filtration with sterile 0.45 μm cellulose membrane filter (Sartorius Stedim Biotech, Germany) was performed by taking 100 μl of the enriched sample in Bolton broth. The filters were placed in small petri dishes (60 × 15 mm) containing 10 ml of Campy-Cefex medium supplemented with 5% horse blood in small 60 × 15 mm petri dishes containing 10 ml of this medium. The samples were passively filtered for 15–20 min at room temperature. Initially, tests were performed withthe filter left in place during incubation and in other cases removing it at 15–20 min. However, it was decided to remove the filter after 15–20 min, because that better results were obtained by finding more isolated colonies of *Campylobacter* spp. The use of filters was performed to increase the recovery of bacterial isolates from the enrichment broth and to reduce competing bacteria [36,37]. The second procedure consisted of taking 10 μl of the enrichment in Bolton broth with a sterile plastic loop and plating by depletion in Campy-Cefex culture medium. The boxes of both procedures were incubated under microaerobiosis conditions using the CampyGen Oxoid® envelope at 42 °C for 24 ± 2 h. Then, passages were made on Campy-Cefex and Karmali media (Liofilchem®, Italy) to obtain pure cultures of presumptive *Campylobacter* colonies (Fig. S1, Supplementary Material). Colonies were subjected to Gram staining, a motility test in 0.85% saline, and oxidase and catalase tests were performed. Confirmed isolates were stored at −20 °C in 10% Skim Milk broth (Merck, Darmstadt Germany).

## 3 Results

### 3.1 Prevalence

Of the 91 samples processed, 42 were positive for *Campylobacter* spp., and the prevalence was 46.2% (42/91). Of the 42 positive samples, *C. jejuni* was the most prevalent species with 54.8% (23/42) positive samples, followed by co-contamination of *C. jejuni* and *C. coli* in the same sample, 35.7% (15/42) and a small percentage of samples were contaminated only with *C. coli* 9.5% (4/42). The associations between the prevalence of *Campylobacter* in retail chicken carcasses and the place of origin (farmers' markets/small food stores) were statistically significant (p < 0.05) and it was evident that the prevalence was higher in small food stores than in farmers' markets. We also observed that,the species *C. jejuni* was the most common in both farmers' markets and small food stores (Table 1).

**Table 1:** Prevalence of *C. jejuni* and *C. coli* in retail chicken carcasses of farmers’ markets y small food stores.

Location	*C+ (%)*^a^	*C. jejuni* (%)	*C. coli* (%)	*C. jejuni* + *C. coli (%)*^b^
***Farmers' markets***	18/53 (34%)	10/18 (55.5%)	1/18 (5.6%)	7/18 (38.9%)
***Small food stores***	24/38 (63.2%)	13/24 (54.2%)	3/24 (12.5%)	8/324 (33.3%)

a Number of positive samples for *Campylobacter* spp. in farmers' markets was 18 and in small food stores was 24.

b Percentage of positive samples with the joint presence of *C. jejuni*/*C. coli*.

**Fig. 2:**
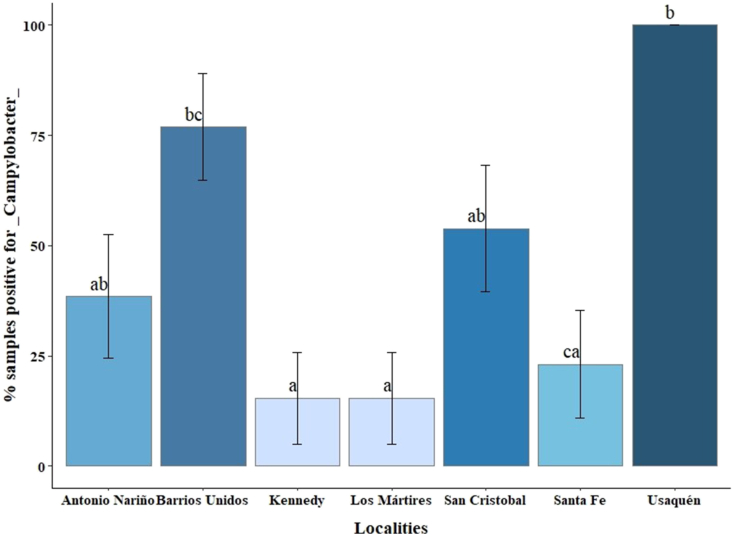
Percentage of positive samples for *Campylobacter* in seven localities of the city of Bogotá. Different letters indicate statistically significant differences between localities (p < 0.1). Bars indicate ± standard error (n: 13 per locality).

Regarding the localities, Usaquén had the highest prevalence (100%), followed by Barrios Unidos (76.9%), San Cristóbal (53.9%), and Antonio Nariño (38.5%). The lowest prevalence of *Campylobacter* was recorded in Santa Fe (23.1%), Kennedy (15.4%), and Los Mártires (15.4% each). The association between the prevalence of *Campylobacter* spp. In retail chicken carcasses and the locality of origin was significant (p < 0.001). To show the differences in *Campylobacter* prevalence among the localities, data were analyzed using the GLM model and it was found that the locality of Usaquén presented significant differences (p < 0.1) with the localities of Santa Fe, Kennedy and Los Mártires (Fig. 2). On the other hand, Table 2 shows the prevalence of the species found by locality, where the Barrios Unidos locality was significantly different from Kennedy and Los Mártires (p < 0.1). In addition, the Barrios Unidos locality was the only one that presented a prevalence for *C. jejuni* (50%), *C. coli* (20%) and simultaneous *C. jejuni*/*C. coli* contamination (30%).

**Table 2:** Prevalence of *C. jejuni* and *C. coli* species in retail chicken carcasses in the different localities sampled in the city of Bogotá.

Localities	*C+ (%)*^a^	*C. jejuni* (%)	*C. coli* (%)	*C. jejuni* + *C. coli (%)*^b^
**Antonio Nariño**	5/13 (38.5%)	4 (80%)	0 (0%)	1 (20%)
**Barrios Unidos**	10/13 (76.9%)	5 (50%)	2 (20%)	3 (30%)
**Kennedy**	2/13 (15.4%)	2 (100%)	0 (0%)	0 (0%)
**Los Mártires**	2/13 (15.4%)	1 (50%)	0 (0%)	1 (50%)
**Usaquén**	13/13 (100%)	3 (23.1%)	0 (0%)	10 (76.9%)
**Santa Fe**	3/13 (23.1%)	2 (66.7%)	1 (33.3%)	0 (0%)
**San Cristóbal**	7/13 (53.9%)	6 (85.7%)	1 (14.3%)	0 (0%)

a The total number of samples positive for *Campylobacter* spp.

b Percentage of positive samples with the joint presence of *C. jejuni*/*C. coli*.

In addition, the localities of Antonio Nariño, Los Mártires, Usaquén and San Cristóbal presented contamination with samples containing *C. jejuni* and others with simultaneous *C. jejuni*/*C. coli* contamination, but none presented only positive samples with *C. coli*. On the contrary, samples from the Santa Fe locality did not present simultaneous contamination with *C. jejuni/C. coli*, but separately with *C. coli*, and samples from the Kennedy locality were the only ones that showed contamination with *C. jejuni* only (Table 2). It was also shown that *C. jejuni* was the species with the highest prevalence compared to *C. coli* in each locality and that the samples from the Kennedy locality were the only ones that presented contamination only with *C. jejuni* compared to the other localities that presented contamination with the both species of *Campylobacter* spp. It also shows an important prevalence of the co-occurrence presence of *C. jejuni/C. coli* in the Usaquén locality.

Regarding the results obtained in each locality according to the place of origin (farmers' markets/small food stores), it was found that in most cases the highest percentage of contaminated samples came from small food stores in most cases and again we found that *C. jejuni* was the most common species in both farmers' markets and small food stores in each of the localities (Table 3).

**Table 3:** Prevalence of *Campylobacter* spp. in chicken carcasses from farmers' markets and small food stores in different localities.

Localities	Location	n^a^	*C. jejuni* (%)	*C. coli* (%)	*C. jejuni* + *C. coli (%)*^b^
**Antonio Nariño**	Farmers' markets	1	100	0	0
	Small food stores	4	75	0	25
**Barrios Unidos**	Farmers' markets	5	80	0	20
	Small food stores	5	20	40	40
**Kennedy**	Farmers' markets	0	0	0	0
	Small food stores	2	100	0	0
**Los Mártires**	Farmers' markets	1	100	0	0
	Small food stores	1	0	0	100
**Usaquén**	Farmers' markets	8	25	0	75
	Small food stores	2	0	0	100
**Santa Fe**	Farmers' markets	0	0	0	0
	Small food stores	2	50	50	0
**San Cristóbal**	Farmers' markets	2	50	50	0
	Small food stores	5	100	0	0

a The total number of samples positive for *Campylobacter* spp.

b Percentage of positive samples with the joint presence of *C. jejuni*/*C. coli*.

### 3.2 Risk factors

This study identified four (4) possible risk factors associated with *Campylobacter* contamination in chicken meat marketed in retail outlets in the city of Bogotá. It is important to note that taking these factors together could explain most of the contamination. The significant factors that were poor cleaning of the scales (OR: 41.42, 95% CI: 7.57–226.6, p = < 0.001), low frequency of disinfection of in food handling utensils (OR: 45.94, 95% CI: 5.4–392.9, p = < 0.001), type of place (farmers' markets/small food stores) (OR: 4.24, 95% CI: 1.29–13.9, p = 0.0169), direct contact of chicken carcasses with other poultry meat food or prey (OR: 20.59, 95% CI: 4.03105, p = 0.001) (Table 4). The above indicates that the probability of cross-contamination with *Campylobacter* due to poor scale cleaning and low frequency of disinfection higher as result of contact of chicken carcasses with other food and originating from small food higher than the probability of contamination as stores/farmers' markets.

**Table 4:** Conditional logistic regression analysis for possible risk factors related to the prevalence of *Campylobacter* in retail chicken carcasses in farmers' markets and small food stores in the different localities sampled in the city of Bogotá.

Risk Factor Variables	Category	% (No. positives/observations^a^) positives	P value	OR	CI 95%	
**Use personal protective equipment**	Good	47.1% (16/34)	REF	NA	NA	NA
	Regular	70.6% (12/17)	0.12	2.64	0.77	9.05
**Frequency of utensil disinfection**	Each time they are used	6.3% (1/16)	REF	NA	NA	NA
	Twice a day	77.1% (27/35)	<0.001^b^	45.94	5.37	392.9
**Sample temperature at the time of purchase**	Within cooling range 0–4 °C	50% (16/32)	REF	NA	NA	NA
	Greater than cooling range 0–4 °C	63.2% (12/19)	0.368	1.69	0.53	5.35
**Type of location**	Farmers' markets	39.3% (11/28)	REF	NA	NA	NA
	Small food stores	73.9% (17/23)	0.0169^b^	4.24	1.29	13.9
**Origin of chicken**	Food plant	56.1% (23/41)	REF	NA	NA	NA
	Farm	50% (5/10)	0.731	0.78	0.19	3.09
**Contact of chicken carcasses with other food**	No	30% (9/30)	REF	NA	NA	NA
	Yes	90.5% (19/21)	0.001^b^	20.59	4.03	105
**The scale is for the exclusive use of the chicken**	Yes	50% (18/36)	REF	NA	NA	NA
	No	66.7% (10/15)	1.973	0.50	0.56	6.84
**The scale is cleaned each time it is used**	Yes	10% (2/20)	REF	NA	NA	NA
	No	83.9% (26/31)	<0.001^b^	41.42	7.57	226.6

a The total number of observations analyzed for each risk factor variable was 5.

b Significance (p < 0.05), NA: Not Applicable, REF: Reference Category.

## 4 Discussion

In Colombia, there have been no studies to compare the prevalence of *Campylobacter* in chicken carcasses sold at retail in the country or Bogotá, so this is the first study with this objective. Bogotá is the largest market city in the country, contributing more than a third of the gross domestic product (GDP) (31%) and is considered an engine of the national economy due to the size of its population, being the most populated city in the country, with 7.8 million according to the latest census conducted by the National Administrative Department of Statistics (DANE) in 2018 [38]. Its inhabitants represent approximately 15.4% of the national population [39,40]. In terms of chicken production, the department of Cundinamarca (of which Bogotá is part) ranks second among the departments with the largest share in the country's poultry sector with 20.2% of GDP [39].

The overall prevalence of *Campylobacter* obtained in this study was 46.2% in chicken carcasses sold in retail establishments in Bogotá. Since we do not have data on the epidemiology of this bacterium in Colombia, we do not know whether this scenario is recent or long-standing. However, when comparing the results of this study with data from other international investigations, it is evident that the prevalence of *Campylobacter* in raw chickens sold at retail was higher (46.2%) but close to those reported in American countries such as Argentina 32% [41], Brazil 35.8% [42], Ecuador 44% [43], Mexico 43% [36] and then in European countries such as Spain 39.4% [44], Italy 34.1% [45], or Asian countries such as China 45.1% [17] and Pakistan 35.2% [46]. Nevertheless, higher prevalence values close to those reported in this study are found in other countries: such as the United States 59.2% [47], Thailand 57% [48] and Western Australia 58.7% [49] Haz clic o pulse aquí para escribir texto.The reported differences in prevalence among these studies may be due to possible differences in the sampling scheme or design, the number of samples analyzed, the type of sample (fresh versus frozen chicken), the detection protocol, chicken production systems, among others.

Furthermore, *C. jejuni* was found to be the most frequent species compared to *C. coli* and a significant proportion of contamination by both species (*C. jejuni/C. coli*) was evident. In addition, interesting results of joint contamination by both species (*C. jejuni/C. coli*) were found. The above is consistent with what has been observed in several studies showing that *C. jejuni* is more prevalent than *C. coli* in the contamination of chicken carcasses at retail level [15,41,42,48,50,51]. The high prevalence of *C. jejuni* in chicken carcasses may be due the fact that this bacterial species usually has a higher survival rates at low temperatures compared to *C. coli* [48,52]. However, further studies are needed to compare the prevalence and survival kinetics of these bacterial species in chickens.

There were differences in the proportion of chicken carcasses contaminated with *Campylobacter* spp. in farmers' markets vs. small food stores. A higher prevalence of the bacterium was found in the latter and *C. jejuni* was found to be the most common species in both establishments. Although in Colombia there is no information on *Campylobacter* spp. in these places, some international studies have obtained similar results regarding a higher prevalence in small food stores than in farmers' markets [48,53]However, other studies have shown a higher prevalence in farmers' markets than in small food stores [[54], [55], [56]], and these differences may be due to the level of sanitation, when handling, processing and performing storage operations in these stores [57]. Therefore, more studies on the contamination of chicken carcasses with *Campylobacter* spp. in this type of retail locations need to be conducted in Colombia for a better comparison.

Regarding the co-contamination of *C. jejuni* and *C. coli* in chicken carcasses, it is emphasized that these results are important because, although *C. jejuni* and *C. coli* have very similar phenotypic and genotypic characteristics, it has been shown that *C. jejuni* has an advantage over *C. coli* in the process of infection in humans because it carries important virulence determinants that are essential to cause its pathogenesis in comparison to *C. coli* [58]. Nevertheless, it has also been shown that *C. coli* infection was found in older people compared to *C. jejuni* and there is also evidence of greater resistance to various antibiotics such as erythromycin in *C. coli* than in *C. jejuni* [59,60]. These two species are among the top four causes of diarrheal disease worldwide [61]. Therefore, it is important to conduct studies to analyze the possible public health implications of finding foods, such as chicken, contaminated with both species at the same time.

Moreover, this study's shows notable differences in the prevalence percentages of the species of *C. jejuni* and *C. coli* in each locality. In the locality of Usaquén, we observed that all the samples obtained were contaminated with *Campylobacter* spp. This is an important finding since this locality has one of the three main food supply centers in Bogotá: the Codabas farmers' market, which supplies part of the northern part of the city. Approximately 70% of the food consumed by the inhabitants of the surrounding communities comes from this food distribution center [62]. Likewise, the locality of Barrios Unidos presented a high prevalence (76.9%) of contamination with *Campylobacter* spp. This locality borders the locality of Usaquén and possibly the chicken retail sites such as the small food stores in this locality may be supplied by the Codabas farmers' markets, so there may be a flow in the contamination of chicken carcasses in this locality when purchased in places that present presumptive contamination with *Campylobacter*, such as Codabas.

However, the other sampled localities showed low prevalence percentages, with exception of the locality of San Cristóbal. These localities are apparently supplied with poultry products by the farmers' markets supply corporation (Corabastos) of the locality of Kennedy, a farmers' markets where all the products from the different regions of Colombia arrive [39,40,63]. In the locality of Kennedy, two positive samples were obtained that came from small food stores and not from the farmers' markets, which leads us to consider that possibly, in this central supply center, adequate control and hygiene practices are being maintained with respect to this pathogen. However, it is necessary to carry out further studies with a larger number of samples and locations to obtain more information on the prevalence of *Campylobacter* in these localities.

The percentage prevalence of *Campylobacter* evidenced in this study in retail chicken channels in Bogotá contributes to having more information about this pathogen in order to generate improvements in hygienic practices and strengthen training programs for retail establishments to reduce the presence of *Campylobacter* in foods such as raw chicken [48]. The risk factors found in this study are consistent with those of other studies [[64], [65], [66]], which evidence that poor utensil and equipment hygiene are associated with a high incidence of *Campylobacter* species isolated from chicken meat. A possible reason for these findings is the poor use of disinfection protocols and, the use of disinfectant concentrations below the recommended level, inadequate exposure time, among other variables that may influence the efficacy of disinfectants against *Campylobacter* [67,68]. Some disinfectants, such as chlorine and iodine releasers, are microbicidal halogens that are widely used for disinfectant purposes on bacteria such as *Campylobacter* [67]. One of the most used according to the respondents is sodium hypochlorite, but in the study by Gutiérrez-Martín et al., 2011 [67] they evidenced that this disinfectant may be ineffective against *C. jejuni*. However, other studies have obtained good results against *Campylobacter* when using 0.63% sodium hypochlorite, is used and left on for 5 min [69]. Considering these studies, a longer exposure time and/or a higher concentration might make the disinfectant more effective. Therefore, it is advisable to develop studies to evaluate the effectiveness of the cleaning and disinfection protocols against *Campylobacter* spp. being used in the poultry industry and in places where chicken is sold at retail.

These results can be helpful for the surveillance of *Campylobacter* spp. in Bogotá and to promote the initiative to carry out more studies in other cities of the country. They are also important data for the formulation of policies to further strengthen the food safety system along the chain of production, processing, and mainly commercialization of retail poultry. Therefore, it is important to establish strategies to reduce the prevalence of *Campylobacter* in raw chicken and reduce the risk of a possible disease or condition such as campylobacteriosis. Implementation of good agricultural and management practices improved hygienic conditions and consumer education on food safety when handling raw poultry can help reduce the contamination of poultry products with this bacterium. Likewise, the creation and implementation a manual of good hygienic practices for the sale of poultry at farmers' markets and small food stores can be a strategy to educate and raise awareness among vendors and consumers regarding the proper handling of poultry carcasses. Most of the risk factors were associated with poor hygienic and disinfection practices, so, to improve these conditions in retail outlets, personnel should be informed and sensitized about the risk of this pathogen, especially regarding food safety and its relevance mainly in chicken meat.

## 5 Conclusions

This study is the first report on the prevalence of *Campylobacter* in fresh chicken carcasses for human consumption in small food stores and farmers' markets in Bogotá. The results obtained indicate that *C. jejuni* is the most predominant species, followed by *C. coli.* Furthermore, inadequate cleaning of equipment and utensils; and reduced frequency of cleaning and disinfection are possible potential risk factors for the presence of *Campylobacter* in chicken carcasses marketed in the city, highlights the importance of strengthening cleaning and disinfection systems in retail outlets such as farmers' markets and small food stores. Therefore, these results can be helpful for the surveillance of *Campylobacter* spp. in Colombia and for implementation surveillance programs for *Campylobacter* spp. to control its presence in products offered to consumers. Likewise, the proposal of quality assurance strategies and the creation and implementation of a manual of good hygiene practices for the sale of chicken in farmers' markets and small food stores at retail level can be way to educate and raise awareness among sellers and consumers regarding the proper handling of chicken. It is important to highlight the need to carry out more studies to determine the general prevalence of *Campylobacter* spp. in chicken meat intended for human consumption in the country, thus having an approach to the actual situation of this pathogen in Colombia, which will allow the regulatory authorities to establish the necessary measures to reduce a possible impact of this pathogen on public health and also to generate education among consumers for the proper handling and preparation of this food at home.

## Funding

This research did not receive any specific grant from funding agencies in the public, commercial, or not-for-profit sectors.

## Ethical considerations

This study was reviewed and approved by Ethics Committee Faculty of Engineering, Universidad de los Andes, with the approval number: 241. All participants provided informed consent to participate in the study and for the publication of their anonymized case details.

## Data availability statement

The original contributions presented in the study are included in the article/supplementary materials, further inquiries can be directed to the corresponding author/s.

## CRediT authorship contribution statement

**Brigithe Tatiana Ortiz:** Conceptualization, Data curation, Formal analysis, Investigation, Methodology, Writing – original draft, Writing – review & editing. **Deyci Rodríguez:** Conceptualization, Investigation, Supervision, Validation, Writing – original draft, Writing – review & editing. **Silvia Restrepo:** Conceptualization, Investigation, Supervision, Validation, Writing – original draft, Writing – review & editing, Resources.

## Declaration of competing interest

The authors declare that they have no known competing financial interests or personal relationships that could have appeared to influence the work reported in this paper.
