# Supplementary material for: Prevalence and risk factors of Campylobacter jejuni and Campylobacter coli in fresh chicken carcasses from retail sites in Bogotá, Colombia
Source: Heliyon. 2024 Feb 17;10(4):e26356. doi: 10.1016/j.heliyon.2024.e26356 (PMC10900410; doi:10.1016/j.heliyon.2024.e26356)

Supplementary Material

Table S1- Summary of the variables investigated as possible risk factors associated with *Campylobacter* contamination of raw chicken in retail outlets (farmers' markets and small food stores) in Bogotá.

Variables.	Categorization and description
**1. Use of work clothes**	▪ Yes<br>▪ No
**2. Use protective elements**	▪ Good (5 points)<br>▪ Acceptable (4-3 points)<br>▪ Bad (2- 0 points)<br>▪ Hand gloves = 2<br>▪ White uniform= 1<br>▪ Mouthpiece=1<br>▪ Cap/coif=1
**3. Type of worktop top surface**	▪ Wood<br>▪ Stainless steel<br>▪ Marble<br>▪ Others
**4. Use of disinfectants to clean facilities**	▪ Yes<br>▪ No
**5. Frequency of disinfection of utensils used for food handling**	▪ Each time it is used<br>▪ Once a day<br>▪ Two or more times a day<br>▪ Do not disinfect
**6. Meat display mode at the counter**.	▪ Closed refrigerator<br>▪ Open cooler<br>▪ Container with ice without lid<br>▪ Ice container with lid
**7. Sample temperature at the time of purchase**	▪ Within cooling range 0 - 4 °C<br>▪ Greater than cooling range 0 - 4 °C<br>▪ Lower than cooling range 0 - 4 °C
**8. Type of location**	▪ **Farmers' markets**<br>Retail chicken outlets that are within a commercial farmers' market, which included small food stores selling meat, as well as small food stores selling fruits and vegetables and selling raw chicken.<br>▪ **Small food stores**<br>Butcher shops, company-owned small food stores or small neighborhood small food stores selling raw chicken
**9. Chicken origin**	▪ Farm<br>▪ Food plant
**10. Carcasses are in direct contact with other chicken feed or prey.**	▪ Yes<br>▪ No
**11. If the carcasses are in direct contact with another type of chicken feed or prey, which is it?**	▪ Chicken wings<br>▪ Creole chicken<br>▪ Chicken breast<br>▪ Chicken viscera<br>▪ Chicken thighs<br>▪ Other food

**12. The scale on which the chicken is weighed is for the exclusive use of this food.**	▪ Yes<br>▪ No
**13. The scale on which the food is weighed is cleaned each time it is used.**	▪ Yes<br>▪ No

Figure S1. Colonies of *Campylobacter jejuni* ATCC® 33560 on CampyCefex agar supplemented with 5% horse defibrinated blood (A). Colonies of *Campylobacter jejuni* ATCC® 33560 on Karmali agar (B).

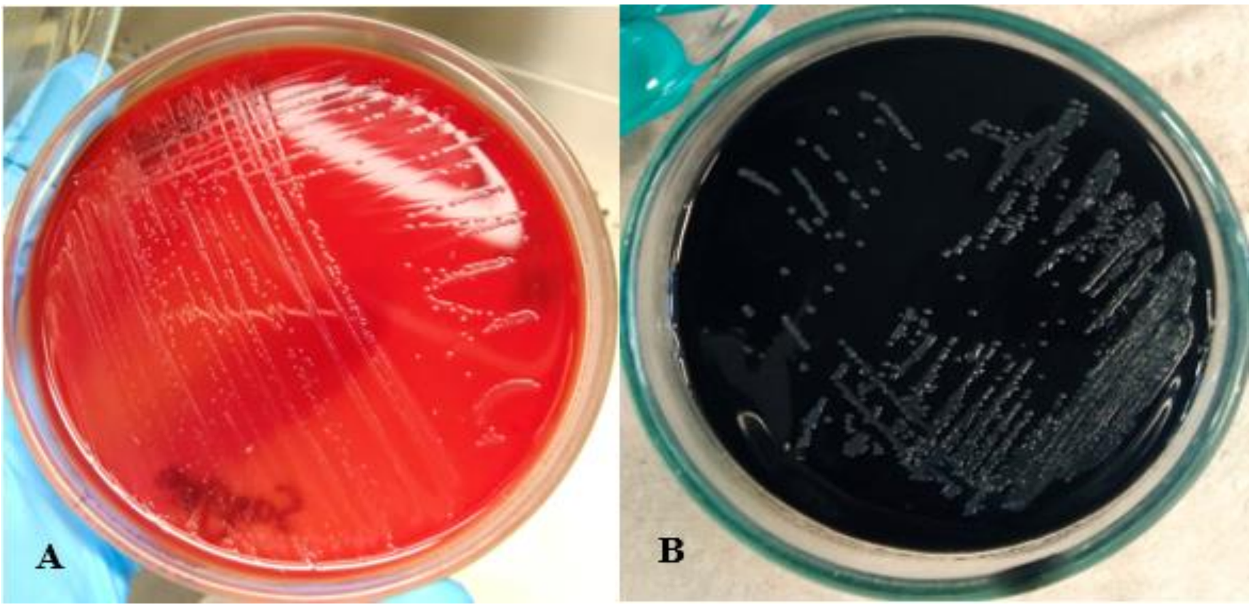

Supplement: Multimedia component 1 [file mmc1.pdf]
